# Constructing Self-Healing Polydimethylsiloxane through Molecular Structure Design and Metal Ion Bonding

**DOI:** 10.3390/polym16101309

**Published:** 2024-05-07

**Authors:** Lvchao Qiu, Yutong Zhou, Zhoufeng Zhao, Qi Wang, Lijun Chu, Shipeng Wen

**Affiliations:** 1 State Grid Zhejiang Electric Power Co., Ltd., Research Institute, Hangzhou 310014, China; 2 State Key Laboratory of Organic-Inorganic Composites, Beijing University of Chemical Technology, Beijing 100029, China

**Keywords:** polydimethylsiloxane, metal coordination bonds, coordination cross-linking network, self-healing capability, mechanical properties

## Abstract

Self-healing polydimethylsiloxane (PDMS) has garnered significant attention due to its potential applications across various fields. In this study, a functionalized modification of PDMS containing di-aminos was initially conducted using 2,6-pyridinedicarbonyl chloride to synthesize pyridine-PDMS (Py-PDMS). Subsequently, rare earth metal europium ions (Eu^3+^) were incorporated into Py-PDMS. Due to the coordination interaction between Eu^3+^ and organic ligands, a coordination cross-linking network was created within the Py-PDMS matrix, resulting in the fabrication of Eu^3+^-Py-PDMS elastomer. At a molar ratio of Eu^3+^ to ligands of 1:1, the tensile strength of Eu^3+^-Py-PDMS reached 1.4 MPa, with a fracture elongation of 824%. Due to the dynamic reversibility of coordination bonds, Eu^3+^-Py-PDMS with a metal-to-ligand molar ratio of 1:2 exhibited varying self-healing efficiencies at different temperatures. Notably, after 4 h of repair at 60 °C, its self-healing efficiency reached nearly 100%. Furthermore, the gas barrier properties of Eu^3+^-Py-PDMS with a molar ratio of 1:1 was improved compared with that of Eu^3+^-Py-PDMS with a molar ratio of 1:1. This study provides an effective strategy for the design and fabrication of PDMS with high mechanical strength, high gas barrier properties, and exceptional self-healing efficiency.

## 1. Introduction

Polydimethylsiloxane (PDMS), renowned for its high flexibility, excellent chemical stability, and weather resistance, is widely utilized across diverse fields including aerospace, healthcare, artificial intelligence, seals, and various other fields [1,2]. However, traditional PDMS typically undergoes permanent cross-linking through chemical covalent bonds [3]. Although covalently cross-linked PDMS exhibits stable properties and good resilience, the irreversible nature of its covalent network in PDMS make it encounter obstacles in restoring its structure post-damage during service, leading to a reduced lifespan and resource wastage. To address this limitation, conferring self-healing capabilities to PDMS emerges a promising alternative strategy.

Present efforts to endow PDMS with self-healing properties involve integrating dynamic bonds, such as dynamic covalent bonds [4,5,6,7,8], hydrogen bonds [9,10,11,12,13], ionic interactions [14,15,16,17], and metal coordination bonds [18,19,20,21,22], into its cross-linking network. Among these, metal coordination bonds stand out as a unique form of non-covalent interaction formed between metal ions and organic molecules, offering high bond energies and design versatility. For instance, Sun et al. [22] grafted carboxyl groups onto polymethylvinylsiloxane (PMVS) chains, creating carboxylated polymethylvinylsiloxane (PMVS-COOH), and introduced FeCl_3_ to facilitate coordination bonds between Fe^3+^ and COO^−^. This complexation, coupled with hydrogen bonds between carboxyl groups, endowed the material with impressive self-healing capabilities. Zhao et al. [23] modified (3-aminopropyl)-terminated PDMS by incorporating salicylaldimine groups through a mild Schiff base reaction, followed by the introduction of Zn^2+^ ions. This approach facilitated synergistic coordination involving nitrogen and oxygen atoms with Zn^2+^ ions. The resulting Zn^2+^-coordinated PDMS exhibited remarkable mechanical properties, achieving an optimized tensile strength of up to 10.0 MPa. Moreover, it exhibited significant self-healing efficiency, reaching approximately 85% at 80 °C over 20 h. Despite advancements in introducing dynamic non-covalent bonds to enhance the self-healing capabilities of PDMS, achieving an optimal balance between mechanical properties and self-healing efficiency remains challenging. Therefore, advancing the design and fabrication of PDMS with superior mechanical resilience and self-healing capacity remains crucial and poses significant ongoing challenges.

Rare earth metals, as important strategic resources, find extensive applications in the metallurgy and petrochemical industries due to their unique properties [24]. Rare earth ions possess distinctive 4f electron orbitals, exhibiting outstanding optical and electromagnetic characteristics, and have been widely applied in the field of polymer materials science [25]. Moreover, rare earth ions can form metal coordination bonds with organic ligands, offering potential for constructing self-healing polymer materials.

In this study, starting from the structural design of polymers, the reactive amino groups in polydimethylsiloxane (PDMS) molecules were leveraged to incorporate pyridine compounds into PDMS chains, yielding pyridine-functionalized PDMS (Py-PDMS). Additionally, rare earth europium ions (Eu^3+^), which have larger ionic cores and high charges compared to other metal ions, were introduced into Py-PDMS, forming coordination interactions between Eu^3+^ ions and electron-donating groups, leading to the construction of a Py-PDMS complex with Eu^3+^ ions serving as cross-linking centers. The study investigated the impact of Eu^3+^ concentration on the structure, mechanical properties, gas barrier properties, and self-healing performance of the complex.

## 2. Experimental Sections

### 2.1. Materials

The bis-aminopropyl-terminated polydimethylsiloxane (NH_2_-PDMS-NH_2_, Mn = 5000) was supplied by Gelest Inc. (Morrisville, PA, USA). 2,6-Pyridinedicarbonyl chloride was acquired from Shanghai Aladdin Bio-Chem Technology Co., Ltd. (Shanghai, China). Triethylamine (Et_3_N) was provided by Shandong Jinyueyuan New Materials Co., Ltd. (Jinan, China). Anhydrous europium nitrate (Eu(NO_3_)_3_) was purchased from Macklin. Dichloromethane (CH_2_Cl_2_) was purchased from Beijing Chemical Factory (Beijing, China).

### 2.2. Synthesis of Py-PDMS

**Figure 1:**
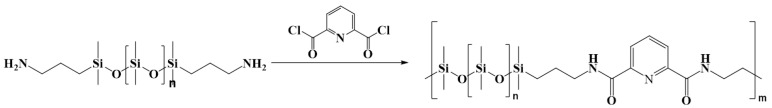
The synthesis route of Py-PDMS.

Firstly, a certain amount of Et_3_N was added to an anhydrous CH_2_Cl_2_ solution of NH_2_-PDMS-NH_2_ under an N_2_ atmosphere at 0 °C. After 2 h of mixing to achieve homogeneity, a solution of 2,6-pyridinedicarbonyl chloride in CH_2_Cl_2_ (with a molar ratio of 2,6-pyridinedicarbonyl chloride to NH_2_-PDMS-NH_2_ of 1:1) was added dropwise using a pressure-equalizing dropping funnel. The resulting mixture was stirred for 2 h at 0 °C under an N_2_ atmosphere, followed by heating the solution to 25 °C and stirring for 48 h. Upon completion of the reaction, the solution was concentrated to 25% of its original volume, followed by coagulation using anhydrous methanol to obtain a viscous white precipitate. The precipitate was then dissolved in CH_2_Cl_2_ and washed repeatedly to remove small molecular by-products. The final product was obtained by vacuum evaporation to remove the solvent and a small amount of Et_3_N, yielding the Py-PDMS. The synthetic route is depicted in Figure 1.

### 2.3. Preparation of Eu^3+^-Py-PDMS Complex

An ethanol solution of anhydrous Eu(NO_3_)_3_ (with the mole ratio of rare earth to ligand determined as 1:1, 1:2, 1:3) was added to the CH_2_Cl_2_ solution of the Py-PDMS prepared in the above step. The mixture was magnetically stirred at 25 °C until no significant stratification was observed after standing. After concentration, the solution was poured into a polytetrafluoroethylene mold with a length of 10 cm and a width of 8 cm, and the solvent was evaporated to form the Eu^3+^ ligand–Py-PDMS (Eu^3+^-Py-PDMS) complex.

### 2.4. Characterization

The molecular structure of Py-PDMS was analyzed using a nuclear magnetic resonance (NMR) spectrometer and a Fourier-transform infrared (FTIR) spectrometer. The number-average molecular weight (Mn) and molecular weight distribution (PDI) of Py-PDMS were determined using an Agilent 1260 gel permeation chromatograph (GPC) with tetrahydrofuran (THF) as the solvent and sample concentrations ranging from 0.01% to 0.05% (*w*/*v*). The thermal stability of Py-PDMS was evaluated using a TGA-1 apparatus manufactured by METTLER TOLEDO, Greifensee, Switzerland. The testing temperature range was 40–800 °C, with a heating rate of 10 K/min under a nitrogen atmosphere. UV–visible spectroscopy analysis was conducted using a Lambda 950 UV–NIR spectrometer, with a sample concentration of 5 μg/mL and a spectral range of 200–800 nm. Differential scanning calorimetry (DSC, METTLER TOLEDO, Switzerland) was used to measure the glass transition temperature (Tg) of Py-PDMS and Eu^3+^-Py-PDMS complexes. The mechanical properties of the Eu^3+^-Py-PDMS complexes with a dumbbell-shaped configuration and a thickness of 1 mm were assessed using a Zwick/Roell Text-port II electronic tensile tester in accordance with the ISO 37:2017 standard [26], employing a tensile speed of 500 mm/min. The self-healing properties of the samples was evaluated based on self-healing efficiency. The testing method involved cutting the samples into two pieces in the middle of the dumbbell-shaped specimens, followed by promptly reattaching the cut surfaces. After healing at different temperatures of 20 °C, 40 °C, and 60 °C for 4 h, the stress–strain properties of these samples were then retested. The self-healing efficiency was quantified as the ratio of the elongation after healing to the original elongation. Morphologies of the repaired samples was observed using a Zeiss Axio Lab 5 polarizing microscope. The nitrogen permeability test was performed on a VAC-V2 gas permeability analyzer (manufactured by Jinan Languang Electromechanical Technology Co., Ltd., Jinan, China) using a manometric method following the ISO 2782-1:2022 standard [27].

## 3. Results and Discussion

### 3.1. Molecular Structure of Py-PDMS

**Figure 2:**
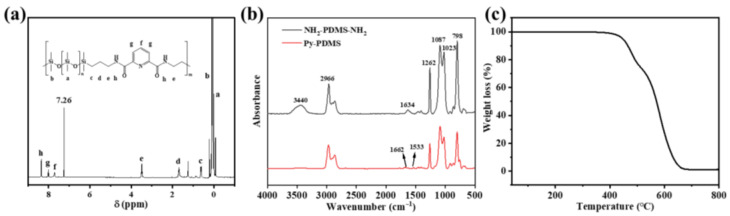
(**a**) ^1^H NMR of Py-PDMS; (**b**) FTIR spectra of NH_2_-PDMS-NH_2_ (black line) and Py-PDMS (red line); (**c**) TGA curve of Py-PDMS.

Figure 2a presents the ^1^H NMR spectra of Py-PDMS. In Figure 2a, the peak observed at 7.26 ppm is attributed to the solvent. Notably, the peak at 8.36 ppm (peak h) corresponds to the H on the amide resulting from the reaction between -NH_2_ and the acyl chloride group. Peaks at 8.02 ppm (peak g) and 7.71 ppm (peak f) signify the H on the pyridine ring of the 2,6-pyridinedicarbonyl chloride. Additionally, peaks at 3.49 ppm (peak e), 1.68 ppm (peak d), and 0.63 ppm (peak c) are associated with the H on the -CH_2_- of the PDMS amino propyl moiety. Peaks a and b are attributed to the H on the side methyl groups of the Py-PDMS main chain.

The molecular structure was further characterized by the FTIR, as shown in Figure 2b. In the spectrum of NH_2_-PDMS-NH_2_, the peaks at 3440 cm^−1^ and 1634 cm^−1^ were assigned to the stretching vibration and scissoring vibration of -NH_2_, respectively. The peak at 2966 cm^−1^ represented the stretching vibration of C-H in methyl groups [28]. The peaks ranging from 1087 to 1023 cm^−1^ corresponded to the stretching vibration of Si-O-Si, while the peak at 1256 cm^−1^ corresponded to the bending vibration of Si-O-Si. Additionally, the peak at 798 cm^−1^ corresponded to the stretching vibration of Si-CH_3_. As for Py-PDMS, the peaks at 3440 cm^−1^ and 1634 cm^−1^ corresponding to the -NH_2_ group disappeared, while a weak new peak emerged at 1533 cm^−1^, indicating the bending vibration of N-H in the amide group. Meanwhile, a weak new peak appeared at 1662 cm^−1^, corresponding to the stretching vibration of C=O. These changes fully demonstrated the successfully synthesis of Py-PDMS. Meanwhile, the Mn of the resulting Py-PDMS was determined to be 76,890, with a PDI of 1.31, as determined by GPC analysis. The TGA curve of the Py-PDMS in Figure 2c reveals a T_5%_ of 445 °C, indicating its excellent thermal stability.

### 3.2. Structure Characterization of Eu^3+^-Py-PDMS

**Figure 3:**
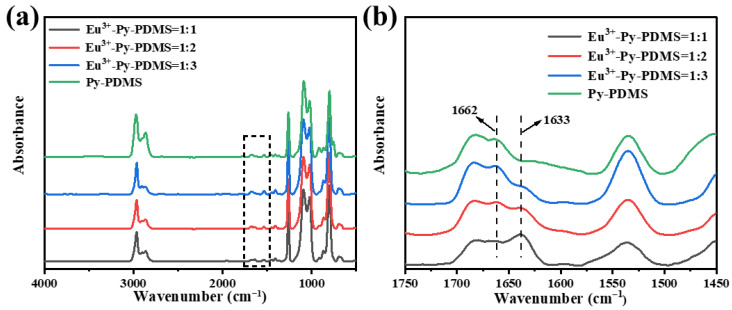
(**a**) FTIR spectra and (**b**) partial amplification spectra of Py-PDMS (green line) and Eu^3+^-Py-PDMS with mole ratios of rare earth to ligand of 1:1 (black line), 1:2 (red line), and 1:3 (blue line).

Figure 3a shows the FTIR spectra of the Eu^3+^-Py-PDMS, with an enlarged view presented in Figure 3b. Upon the incorporation of Eu^3+^, notable changes in peak intensities were detected at 1662 cm^−1^ and 1633 cm^−1^. As analyzed in Section 3.1, the peak at 1662 corresponded to the C=O group. According to reference [29], the emergence of a new peak at 1633 cm^−1^ was attributed to the stretching vibration of C=O-Eu. As the molar ratio of Eu^3+^ to ligand increased from 1:3 to 1:1, the intensity of C=O peak gradually weakened until diminishing, while that of C=O-Eu peak steadily strengthened. These shifts in peak intensities suggested the coordination interaction between Eu^3+^ ions and the ligand of C=O groups in Py-PDMS. More importantly, such coordination interactions were observed to strengthen progressively with higher concentrations of Eu^3+^.

**Figure 4:**
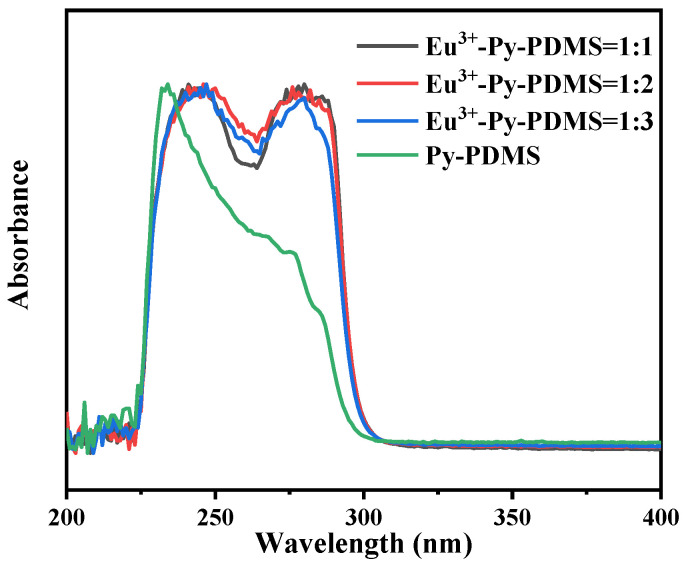
UV spectra of Py-PDMS (green line) and Eu^3+^-Py-PDMS with mole ratios of rare earth to ligands of 1:1 (black line), 1:2 (red line), and 1:3 (bule line).

In Figure 4, the UV spectra of Py-PDMS exhibited a shoulder band at 233 nm, which was absent in Eu^3+^-Py-PDMS. Instead, two new shoulder peaks emerged at 244 nm and 278 nm. This phenomenon can be attributed to metal-to-ligand charge transfer interactions between Eu^3+^ and C-N and C=O bonds present in the Py-PDMS molecular chain. This change indicated a significant alteration in the electronic structure due to the incorporation of Eu^3+^, suggesting potential modifications in the chemical environment and bonding interactions within the complex.

**Figure 5:**
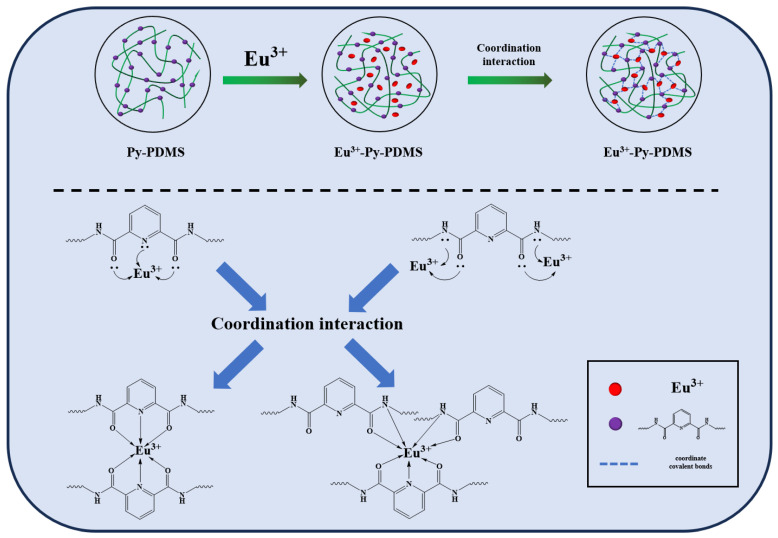
Schematic of the coordination interaction between Eu^3+^ and Py-PDMS.

Based on the analysis of FTIR and UV spectra, the coordination cross-linking network in Py-PDMS is formed by the coordination interaction between metal ions capable of accepting lone pair electrons and ligands capable of donating electrons. The rare earth Eu^3+^ ions possess abundant electron transition energy levels and readily form coordination bonds with Py-PDMS ligands, resulting in the formation of rare earth–polymer complexes. Upon introducing Eu^3+^ into the Py-PDMS matrix, Eu^3+^ coordinated as a center with C-N and C=O in Py-PDMS to generate the Eu^3+^-Py-PDMS complex, as illustrated in Figure 5. This coordination interaction between Eu^3+^ and the ligands within the Py-PDMS matrix enhanced the structural integrity and properties of the resulting complex.

**Figure 6:**
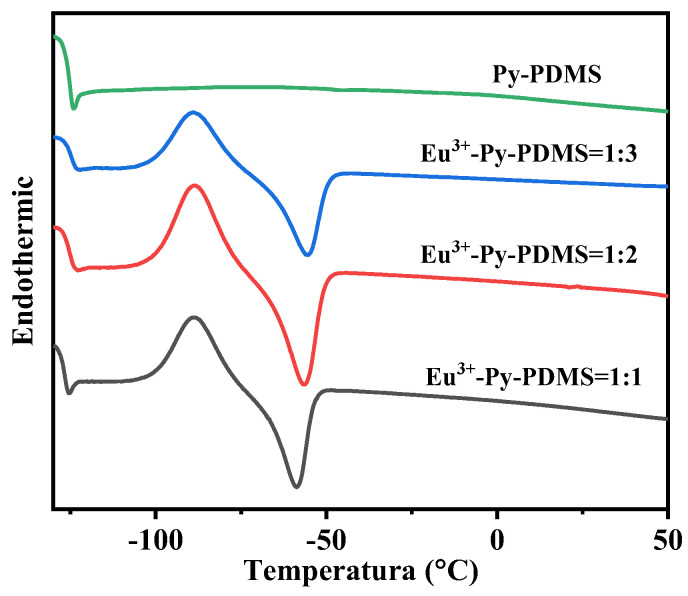
DSC curves Py-PDMS (green line) and Eu^3+^-Py-PDMS with mole ratios of rare earth to ligands of 1:1 (black line), 1:2 (red line), and 1:3 (bule line).

Although the introduction of Eu^3+^ resulted in strong coordination bonds with Py-PDMS ligands, the molecular chains of Py-PDMS still retained great flexibility. As depicted in Figure 6, both Py-PDMS and all Eu^3+^-Py-PDMS complexes exhibited ultra-low glass transition temperatures (Tg) of approximately −125 to −123 °C. Interestingly, all curves of Eu^3+^-Py-PDMS complexes exhibited a melting peak around −50 °C. According to reference [30], this transition temperature indicated the melting of the metal–ligand complex domains. In contrast, this melting peak was not observed in the curve of Py-PDMS. This phenomenon also demonstrated the successful formation of a coordination cross-linking network in Eu^3+^-Py-PDMS.

### 3.3. Mechanical Properties of Eu^3+^-Py-PDMS Complex

**Figure 7:**
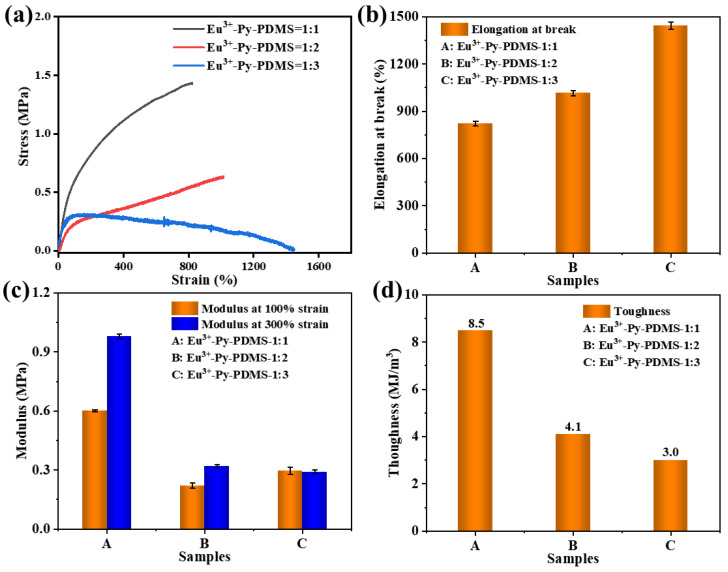
(**a**) Stress–strain curves of Eu^3+^-Py-PDMS; the values of (**b**) elongation at break, (**c**) modulus at 100% and 300% strain, and (**d**) toughness of Eu^3+^-Py-PDMS.

Uncross-linked Py-PDMS exhibits severely limited mechanical properties due to the absence of three-dimensional cross-linking networks. However, the incorporation of Eu^3+^ as a coordination cross-linking center within Py-PDMS confers enhanced mechanical strength and elastomeric characteristic. The stress–strain curves depicted in Figure 7a illustrate the tensile behavior of Eu^3+^-Py-PDMS at varying Eu^3+^ concentrations, and their elongation at break values are displayed in Figure 7b. It is evident that the tensile strength of Eu^3+^-Py-PDMS increased with the increase in Eu^3+^ concentration, while the elongation at break decreased. This trend can be attributed to the heightened presence of Eu^3+^ ions, which facilitated strong interactions between the Eu^3+^ ions and ligands of the Py-PDMS molecular chains. Consequently, strengthened intermolecular interactions between the molecular chains contributed to the observed enhancement in tensile strength. At a molar ratio of 1:1 between Eu^3+^ ions and ligands, Eu^3+^-Py-PDMS achieved a maximum tensile strength of 1.4 MPa while still retaining a substantial elongation at break of 824%.

The stiffness of the elastomers is typically evaluated based on their modulus at 100% and 300% strain. As shown in Figure 7c, there was little difference in the modulus at 100% and 300% strain between Eu^3+^-Py-PDMS with a molar ratio of rare earth to ligands of 1:3 and 1:2. However, a notable increase in the modulus at 100% and 300% strain was observed for Eu^3+^-Py-PDMS with a molar ratio of rare earth to ligands of 1:1. This increment was due to the formation of a more complete coordination cross-linking network at this 1:1 molar ratio. Despite the heightened stiffness of Eu^3+^-Py-PDMS with a molar ratio of rare earth to ligands of 1:1, it still maintained a high level of toughness (which is defined as the area under the stress–strain curve). As depicted in Figure 7d, an increase in the molar ratio of rare earth to ligands resulted in a gradual increase in the toughness of Eu^3+^-Py-PDMS. In comparison to Eu^3+^-Py-PDMS with a molar ratio of rare earth to ligands of 1:3 and 1:2, the toughness of Eu^3+^-Py-PDMS with a molar ratio of rare earth to ligands of 1:1 increased by 183% and 107%, respectively. The observed variation in toughness can be ascribed to the viscoelastic structural changes in Eu^3+^-Py-PDMS with varying coordination cross-linking density. Among these three Eu^3+^-Py-PDMS complexes, the Eu^3+^-Py-PDMS with a molar ratio of rare earth to ligands of 1:2 exhibited moderate mechanical and viscoelastic properties; hence, it was chosen for further exploration of the self-healing capabilities.

**Figure 8:**
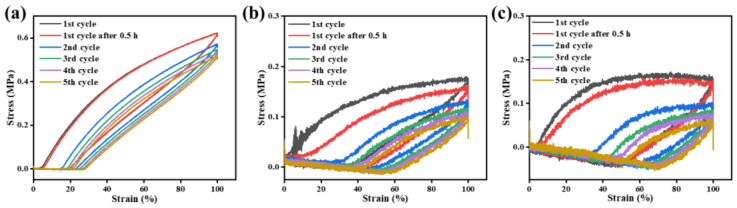
Cyclic stress–strain tests of Eu^3+^-Py-PDMS with a mole ratio of (**a**) 1:1, (**b**) 1:2, and (**c**) 1:3.

The 100% cyclic tensile testing of Eu^3+^-Py-PDMS was further analyzed to investigate the influence of the coordination network formed between Eu^3+^ and Py-PDMS. As shown in Figure 8a–c, Eu^3+^-Py-PDMS with varying concentrations of Eu^3+^ exhibited distinct stress-softening and hysteresis phenomena during cyclic tensile loading, indicative of the viscoelastic characteristics of Eu^3+^-Py-PDMS. With an increase in Eu^3+^ concentration, a reduction in the hysteresis loop was observed. This reduction was attributed to the reinforcement of the coordination network formed by Eu^3+^ at high concentrations, which became less susceptible to disruption during stretching. Consequently, there was a significant increase in the tensile strength of Eu^3+^-Py-PDMS with a molar ratio of 1:1. Additionally, Figure 8 shows that the hysteresis loop of Eu^3+^-Py-PDMS can essentially overlap with the first cycle within 30 min, indicating the reversible recovery of the coordination bonds after fracture.

### 3.4. Self-Healing Properties of Eu^3+^-Py-PDMS Complex

**Figure 9:**
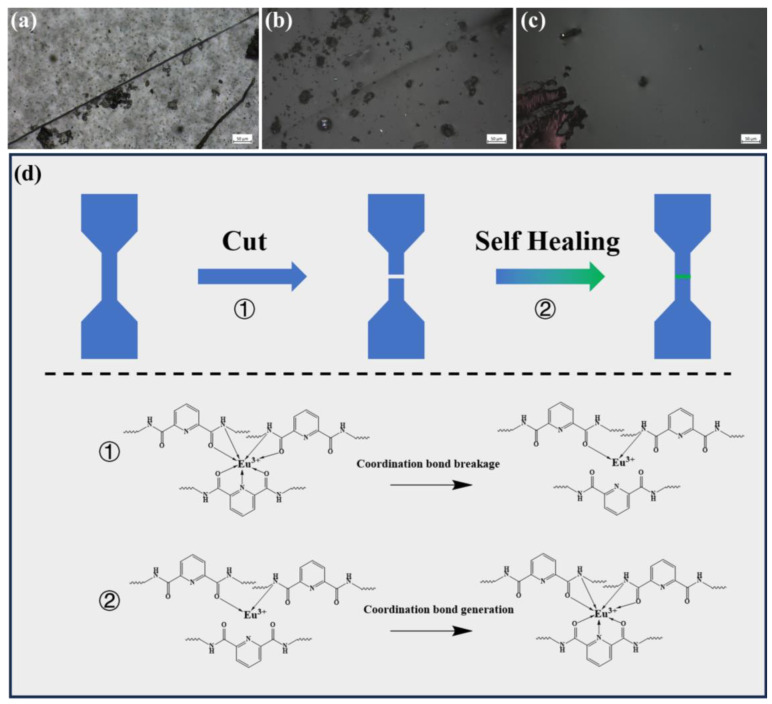
Optical microscope images of Eu^3+^-Py-PDMS with the mole ratio of 1:2 healed at different temperatures for 4 h: (**a**) 20 °C, (**b**) 40 °C, (**c**) 60 °C; (**d**) schematic diagram of the self-healing mechanism of Eu^3+^-Py-PDMS.

The coordination interaction between Eu^3+^ ions and Py-PDMS ligands facilitated dynamic rupture and recovery, thereby granting Py-PDMS with self-healing capabilities. After cutting a slit in the Eu^3+^-Py-PDMS = 1:2 sample with a knife, it was subjected to self-healing for 4 h at temperatures of 20, 40, and 60 °C. The surface morphology of the healed wounds was observed using an optical microscope, as shown in Figure 9a–c. At 20 °C, surface incisions remained visible even after 4 h of self-healing, whereas the incisions disappeared but slight scars persisted after the same duration of repair at 40 °C. Remarkably, at 60 °C, both the incisions and scars completely vanished after 4 h, achieving full self-healing. The self-healing mechanism of Eu^3+^-Py-PDMS is depicted in Figure 9d.

**Figure 10:**
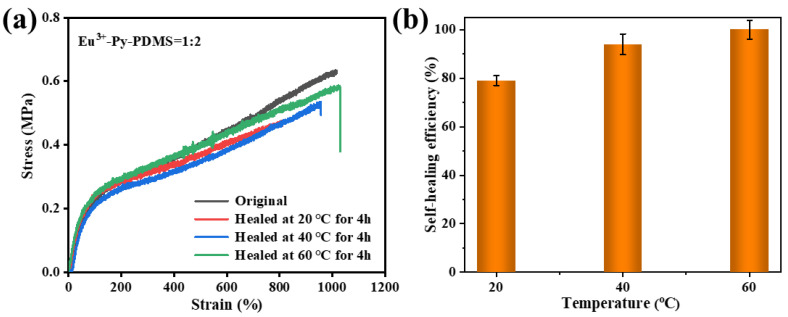
(**a**) Stress–strain curves of Eu^3+^-Py-PDMS with a mole ratio of 1:2 before and after self-healing at different temperatures for 4 h; (**b**) the self-healing efficiency of Eu^3+^-Py-PDMS with a mole ratio of 1:2 at different temperatures for 4 h.

Typically, the self-healing efficiency of elastomers is defined as the ratio of tensile strength or elongation at break before and after repair [31]. Here, the self-healing efficiency was defined as the ratio between the elongation after healing to the original elongation at break. To further investigate the self-healing efficiency of Eu^3+^-Py-PDMS, stress–strain curves were measured after 4 h of self-healing at temperatures of 20, 40, and 60 °C, as illustrated in Figure 10a. It is evident that with increasing repair temperature, the elongation at break of Eu^3+^-Py-PDMS gradually increased. The initial elongation at break of Eu^3+^-Py-PDMS = 1:2 was 1015%. After 4 h of repair at 20 °C, its repair efficiency reached 79%, while at 40 °C and 60 °C, the self-repair efficiencies reached 94% and nearly 100%, respectively (Figure 10b). As the temperature rose, the mobility rate of Py-PDMS molecular chains was accelerated, facilitating the rearrangement of coordination bonds between Eu^3+^ and ligands in the Py-PDMS, thereby reconnecting the Py-PDMS molecular chains and resulting in an enhanced self-healing efficiency of Eu^3+^-Py-PDMS. Compared to other metal-coordinated PDMS, the resultant Eu^3+^-Py-PDMS exhibited superior self-healing performance, as depicted in Table 1.

### 3.5. Gas Barrier Properties of Eu^3+^-Py-PDMS Complex

**Figure 11:**
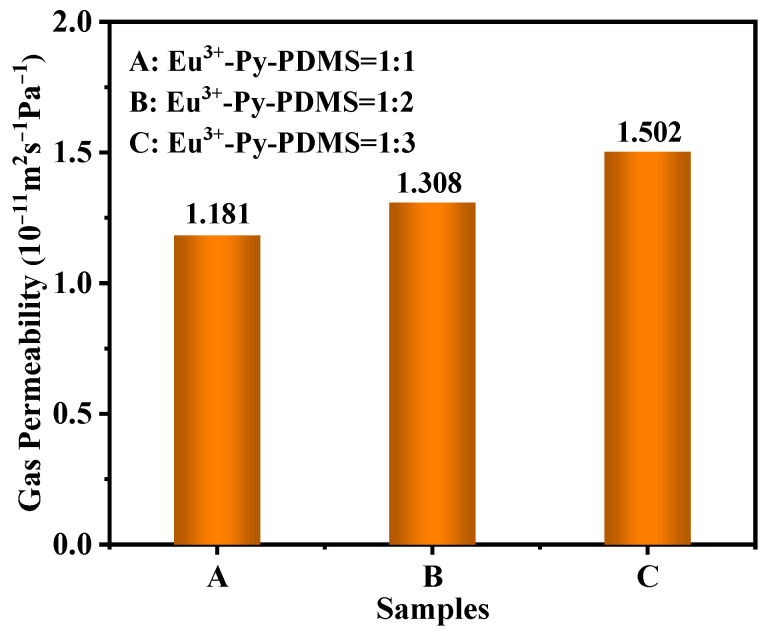
Gas permeability of Eu^3+^-Py-PDMS with different metal-to-ligand molar ratios.

Due to the highly flexible nature of PDMS molecular chains, an abundant free volume exists within the chains, facilitating the easy permeation of gas molecules through the pathways amidst these free volumes. Consequently, PDMS products typically exhibit poor gas barrier properties. To investigate the effect of Eu^3+^ concentrations on the gas barrier properties of the Eu^3+^-Py-PDMS complex, gas permeability tests (nitrogen) were conducted, as shown in Figure 11. Since the pure Py-PDMS without the addition of Eu^3+^ lacked the necessary cross-linking networks and could not be shaped, the gas permeability data of pure Py-PDMS was not displayed. However, it is evident that the gas permeability of Eu^3+^-Py-PDMS gradually decreased with an increase in the Eu^3+^ concentration, indicating an enhancement in gas barrier properties. For instance, comparing with Eu^3+^-Py-PDMS with a molar ratio of 1:3, the Eu^3+^-Py-PDMS with a molar ratio of 1:1 exhibited a 21.4% increase in gas barrier properties. This improvement can be attributed to the higher concentration of Eu^3+^ providing more coordination sites, resulting in the increased formation of coordination bonds. Consequently, there are heightened intermolecular interactions between Py-PDMS molecular chains, leading to a reduction in free volumes. Thus, the incorporation of Eu^3+^ enhanced the gas barrier properties of the Eu^3+^-Py-PDMS complex. This finding may extend the application field of the resultant Eu^3+^-Py-PDMS complex.

## 4. Conclusions

In conclusion, a novel PDMS with high mechanical strength and exceptional self-healing capabilities was fabricated through molecular structure design and a metal ion bonding strategy. The FTIR and UV spectra confirmed the successful formation of coordination bonds between Eu^3+^ and ligands of Py-PDMS. The strong coordination interactions strengthened the mechanical properties of Eu^3+^-Py-PDMS. When the molar ratio between Eu^3+^ and ligands was 1:1, Eu^3+^-Py-PDMS exhibited a tensile strength of 1.4 MPa and a fracture elongation of 824%. Additionally, due to the dynamic reversibility of coordination interactions, Eu^3+^-Py-PDMS with a metal-to-ligand molar ratio of 1:2 exhibited impressive self-healing capabilities, with a self-healing efficiency reaching nearly 100% after being healed at 60 °C for 4 h. Moreover, the Eu^3+^-Py-PDMS with a molar ratio of 1:1 exhibited a 21.4% increase in gas barrier properties compared to Eu^3+^-Py-PDMS with a molar ratio of 1:3. Overall, this study offers a significant advancement in the fabrication of self-healing PDMS, with potential applications in aerospace, artificial intelligence, seals, and various other fields.

## Figures and Tables

**Table 1:** Comparison of the self-healing performance of other metal-coordinated PDMS.

Samples	Self-Healing Condition	Self-Healing Efficiency	Ref.
PDMS-NNN-Zn	12 h at 25 °C	91.3%	[19]
PDMS-PtL	12 h at room temperature	100%	[32]
Ni-Py-PDMS	72 h at room temperature	90.0%	[33]
IMZ-PDMS	31 h at 25 °C	98%	[34]
Zn-IC-PDMS	24 h at room temperature	96%	[35]
PDMS−TDI−Al	36 h at room temperature	90%	[36]
Fe^3+^-Hpdca-PDMS	48 h at 25 °C	90.0%	[37]
PDMS-COOH/Al/NH_2_	8 h at 60 °C	80%	[38]
Eu^3+^-Py-PDMS	4 h at 20 °C	79%	This study
Eu^3+^-Py-PDMS	4 h at 60 °C	Nearly 100%	This study

## Data Availability

Data are contained within the article.
